# Bimodal Seasonality and Alternating Predominance of Norovirus GII.4 and Non-GII.4, Hong Kong, China, 2014–2017^1^

**DOI:** 10.3201/eid2404.171791

**Published:** 2018-04

**Authors:** Martin Chi-Wai Chan, Kirsty Kwok, Lin-Yao Zhang, Kirran N. Mohammad, Nelson Lee, Grace C.Y. Lui, E. Anthony S. Nelson, Raymond W.M. Lai, Ting F. Leung, Paul K.S. Chan

**Affiliations:** The Chinese University of Hong Kong, Hong Kong, China

**Keywords:** age distribution, bimodal seasonality, GII.4, geographic hotspot, Hong Kong, China, non-GII.4, norovirus, surveillance, vaccine strain, viruses

## Abstract

We report emerging subtropical bimodal seasonality and alternating predominance of norovirus GII.4 and non-GII.4 genotypes in Hong Kong. GII.4 predominated in summer and autumn months and affected young children, whereas emergent non-GII.4 genotypes predominated in winter months and affected all age groups. This highly dynamic epidemiology should inform vaccination strategies.

Human noroviruses are the leading cause of foodborne illnesses across all age groups (*1*) and account for nearly one fifth of all acute gastroenteritis (AGE) infections globally (*2*). Human noroviruses have started superseding rotavirus as the most common cause of AGE in children in regions where rotavirus vaccination has been widely and successfully deployed (*3*). This severe disease burden motivates the development of a norovirus vaccine, and children are an important target group because of high incidence (*4*). The norovirus pandemic GII.4 genotype has been associated with most AGE infections since the mid-1990s (*5*) and thus was an important genotype included and tested in norovirus vaccine clinical trials (*6*); however, this historical predominance of norovirus GII.4 was challenged by the recent emergence of epidemic non-GII.4 genotypes in Asia (*7**–**9*). A better understanding of the changing norovirus epidemiology in Asia might inform the current strategy on norovirus surveillance and vaccine development.

## The Study

Hong Kong is a subtropical coastal city in southern China and has a temperate climate with hot summers and dry winters (Köppen-Geiger climate classification “Cwa”). Since March 2014, we have been monitoring virus genotypes in all hospitalized (i.e., severe) norovirus AGE case-patients of all ages diagnosed at the Prince of Wales Hospital of the Chinese University of Hong Kong and have reported the emergence and predominance of 2 previously less common non-GII.4 genotypes, GII.17 in 2014 and GII.2 in 2016 (*8**,**9*). Here we present further analysis of the seasonal dynamics of different norovirus genotypes during a 42-month period from March 2014 through August 2017. We identified norovirus genotypes in 1,100 (88.3%) of 1,246 case-patients by means of partial viral protein 1 Sanger sequencing and genotype assignment using a genotyping tool available through the National Institute of Public Health and the Environment of the Netherlands (http://www.rivm.nl/mpf/typingtool/norovirus). Seven case-patients were co-infected with >1 norovirus genotype. The proportion of GII.4 genotypes was 49.8% and that of non-GII.4 genotypes 50.2%. Overwhelmingly, most norovirus GII.4 belonged to the GII.Pe-GII.4 Sydney variant (512/544; 94.1%; Technical Appendix Figure 1). The recent recombinant GII.P16-GII.4 Sydney that emerged and predominated in the United States during 2016–2017 (*10*) was only detected sporadically in Hong Kong (Technical Appendix Figure 1). The 2 most prevalent norovirus non-GII.4 genotypes were the recently emerged GII.17 (35.9%) and GII.2 (26.0%) (Technical Appendix Figure 1).

We observed a bimodal seasonality of norovirus AGE requiring hospitalization, with periodic oscillation in the age group of admitted case-patients (Figure, panel A). Among the 19 months that norovirus preferentially affected young children <5 years old (as indicated by a monthly median age of case-patients <5 years), 17 (89%) were predominated by GII.4 genotype, whereas among the 23 months that preferentially affected older children and adults, 19 (83%) were predominated by non-GII.4 genotypes (Figure, panel B). By age groups, norovirus GII.4 accounted for most (68.5%) case-patients who were young children <5 years old, whereas norovirus non-GII.4 predominated in all other age groups: 5–17 years (75.7%), 18–40 years (87.0%), 41–65 years (78.6%), and >65 years (63.2%). The median age of case-patients infected with the recently emerged GII.17 and GII.2 was significantly higher than that for those infected with GII.4 (GII.4, 2 years [interquartile range (IQR) 1–4 years]; GII.17, 49 years [IQR 10–72 years]; GII.2, 5 years [IQR 2–22 years]; p<0.0001, Kruskal-Wallis test) (Technical Appendix Figure 2), as reported earlier over a shorter period (*8**,**9*). By season, late summer and autumn peaks were associated with norovirus GII.4, whereas winter peaks were associated with norovirus non-GII.4 (Figure, panel B). Norovirus infections have become equally common during summer and autumn months (52.3% of all infections during June–November) and during winter and spring months (47.7% of all infections during December–May).

**Figure:**
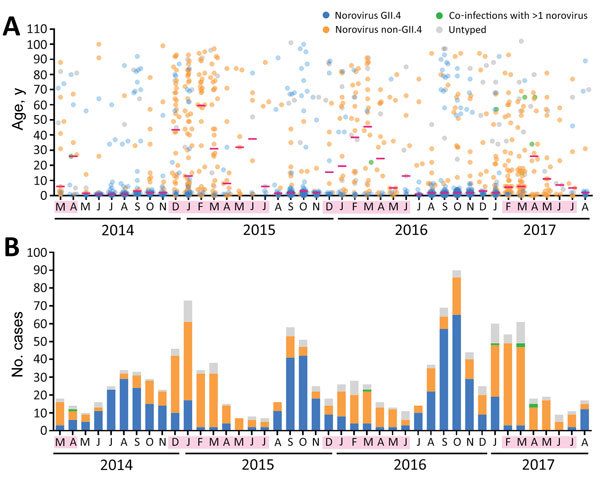
Bimodal seasonality and alternating predominance of norovirus GII.4 and non-GII.4 genotypes in Hong Kong, China, 2014–2017. A) Temporal distribution of ages of patients hospitalized for norovirus gastroenteritis. Each dot represents 1 patient. Red horizontal bars indicate medians. B) Epidemic curve during the study period. All cases shown are stratified by norovirus viral protein 1 genotype. Pink shading along baseline indicates months during which the median age of hospitalized case-patients was >5 years.

## Conclusions

We observed an influenza-like subtropical bimodal seasonality and alternating predominance of norovirus GII.4 and non-GII.4 genotypes, with each infecting different age groups. Norovirus GII.4 predominated in summer and autumn months and preferentially affected young children, who are also one of the age groups most affected by seasonal influenza. In contrast, emergent norovirus non-GII.4 predominated in winter months and affected wider age groups (e.g., all age groups were affected by GII.17 and older children and young adults by GII.2), a pattern which is reminiscent of pandemic influenza viruses. These findings illustrate a highly dynamic epidemiology of norovirus. A similar pattern of alternating epidemics has been observed among the 4 dengue virus serotypes and was shown to reflect moderate but not weak or strong interserotypic cross-protective immunity (*11*). The alternating predominance of norovirus GII.4 and non-GII.4 genotypes in severe infections might reflect an equally complex virus–human immunologic interaction on individual and population levels. This might be explained at least in part by the recently proposed concept that groups norovirus genotypes into so-called immunotypes (*12*). 

The out-of-phase oscillation in the demographic characteristics of norovirus patients admitted to our hospitals complicated clinical resource allocation such as bed management in pediatric and medical wards. High norovirus activity during late summer and autumn overlaps with the local summer peaks of seasonal influenza (*13*) and further increases the burden on the already strained hospital system. We speculate that the recent emergence of non-GII.4 viruses modified the seasonality of noroviruses because a summer peak of this bimodal seasonal pattern occurred only once in an outbreak setting during a 7-year period (2005–2011), during which GII.4 predominated in Hong Kong (http://www.chp.gov.hk/files/pdf/prevention_and_control_of_norovirus_infection_in_hong_kong_r.pdf). 

Hong Kong was implicated as an epicenter in the spread of norovirus GII.17 in China during winter 2014–15 (*14*); whether or not the characteristic bimodal seasonality and alternating epidemic pattern we observed will favor the emergence of new noroviruses would be of public health importance. Of note, alternating predominance of 2 antigenic types of influenza B virus Yamagata lineage has been proposed as a mechanism to generate virus antigenic novelty (*15*). Continued and focused norovirus surveillance and genotyping in Southeast Asia are necessary to identify geographic hotspots of new noroviruses and thus guide vaccine strain selection. The variability of affected age populations among norovirus genotypes underscores the need to consider virus genotype in quantifying norovirus disease burden; this approach will provide more comprehensive evidence to advise future norovirus vaccination strategy.

Technical Appendix Epidemic curve showing bimodal seasonality and alternating predominance of norovirus GII.4 and non-GII.4 and box-plot of age distribution of case-patients infected with norovirus genotypes GII.4, GII.17, and GII.2, Hong Kong, China, 2014–2017.

## References

[1] Havelaar AH, Kirk MD, Torgerson PR, Gibb HJ, Hald T, Lake RJ, et al.; World Health Organization Foodborne Disease Burden Epidemiology Reference Group. World Health Organization global estimates and regional comparisons of the burden of foodborne disease in 2010. PLoS Med. 2015;12:e1001923. 10.1371/journal.pmed.1001923 26633896 PMC4668832

[2] Ahmed SM, Hall AJ, Robinson AE, Verhoef L, Premkumar P, Parashar UD, et al. Global prevalence of norovirus in cases of gastroenteritis: a systematic review and meta-analysis. Lancet Infect Dis. 2014;14:725–30. 10.1016/S1473-3099(14)70767-4 24981041 PMC8006533

[3] Payne DC, Vinjé J, Szilagyi PG, Edwards KM, Staat MA, Weinberg GA, et al. Norovirus and medically attended gastroenteritis in U.S. children. N Engl J Med. 2013;368:1121–30. 10.1056/NEJMsa1206589 23514289 PMC4618551

[4] Lopman BA, Steele D, Kirkwood CD, Parashar UD. The vast and varied global burden of norovirus: prospects for prevention and control. PLoS Med. 2016;13:e1001999. 10.1371/journal.pmed.1001999 27115709 PMC4846155

[5] Siebenga JJ, Vennema H, Zheng DP, Vinjé J, Lee BE, Pang XL, et al. Norovirus illness is a global problem: emergence and spread of norovirus GII.4 variants, 2001-2007. J Infect Dis. 2009;200:802–12. 10.1086/605127 19627248

[6] Bernstein DI, Atmar RL, Lyon GM, Treanor JJ, Chen WH, Jiang X, et al. Norovirus vaccine against experimental human GII.4 virus illness: a challenge study in healthy adults. J Infect Dis. 2015;211:870–8. 10.1093/infdis/jiu497 25210140 PMC5914500

[7] de Graaf M, van Beek J, Vennema H, Podkolzin AT, Hewitt J, Bucardo F, et al. Emergence of a novel GII.17 norovirus – End of the GII.4 era? Euro Surveill. 2015;20:8–15. 10.2807/1560-7917.ES2015.20.26.21178 26159308 PMC5921880

[8] Chan MC, Lee N, Hung TN, Kwok K, Cheung K, Tin EK, et al. Rapid emergence and predominance of a broadly recognizing and fast-evolving norovirus GII.17 variant in late 2014. Nat Commun. 2015;6:10061. 10.1038/ncomms10061 26625712 PMC4686777

[9] Kwok K, Niendorf S, Lee N, Hung TN, Chan LY, Jacobsen S, et al. Increased detection of emergent recombinant norovirus GII.P16-GII.2 strains in young adults, Hong Kong, China, 2016–2017. Emerg Infect Dis. 2017;23:1852–5. 10.3201/eid2311.170561 29048294 PMC5652449

[10] Cannon JL, Barclay L, Collins NR, Wikswo ME, Castro CJ, Magaña LC, et al. Genetic and epidemiologic trends of norovirus outbreaks in the United States from 2013 to 2016 demonstrated emergence of novel GII.4 recombinant viruses. J Clin Microbiol. 2017;55:2208–21. 10.1128/JCM.00455-17 28490488 PMC5483924

[11] Adams B, Holmes EC, Zhang C, Mammen MP Jr, Nimmannitya S, Kalayanarooj S, et al. Cross-protective immunity can account for the alternating epidemic pattern of dengue virus serotypes circulating in Bangkok. Proc Natl Acad Sci U S A. 2006;103:14234–9. 10.1073/pnas.0602768103 16966609 PMC1599940

[12] Parra GI, Squires RB, Karangwa CK, Johnson JA, Lepore CJ, Sosnovtsev SV, et al. Static and evolving norovirus genotypes: implications for epidemiology and immunity. PLoS Pathog. 2017;13:e1006136. 10.1371/journal.ppat.1006136 28103318 PMC5283768

[13] Shu YL, Fang LQ, de Vlas SJ, Gao Y, Richardus JH, Cao WC. Dual seasonal patterns for influenza, China. Emerg Infect Dis. 2010;16:725–6. 10.3201/eid1604.091578 20350403 PMC3321959

[14] Lu J, Fang L, Zheng H, Lao J, Yang F, Sun L, et al. The evolution and transmission of epidemic GII.17 noroviruses. J Infect Dis. 2016;214:556–64. 10.1093/infdis/jiw208 27354370 PMC4957445

[15] Langat P, Raghwani J, Dudas G, Bowden TA, Edwards S, Gall A, et al. Genome-wide evolutionary dynamics of influenza B viruses on a global scale. PLoS Pathog. 2017;13:e1006749. 10.1371/journal.ppat.1006749 29284042 PMC5790164

