# Supplementary material for: Bimodal Seasonality and Alternating Predominance of Norovirus GII.4 and Non-GII.4, Hong Kong, China, 2014–2017
Source: Emerg Infect Dis. 2018 Apr;24(4):767–9. doi: 10.3201/eid2404.171791 (PMC5875276; doi:10.3201/eid2404.171791)
Supplement: Technical Appendix — Epidemic curve showing bimodal seasonality and alternating predominance of norovirus GII.4 and non-GII.4 and box-plot of age distribution of case-patients infected with norovirus genotypes GII.4, GII.17, and GII.2, Hong Kong, China, 2014–2017. [file 17-1791-Techapp-s1.pdf]

Article DOI: https://doi.org/10.3201/eid2404.171791

Bimodal Seasonality and Alternating Predominance of Norovirus GII.4 and Non-GII.4, Hong Kong, China, 2014–2017

Technical Appendix

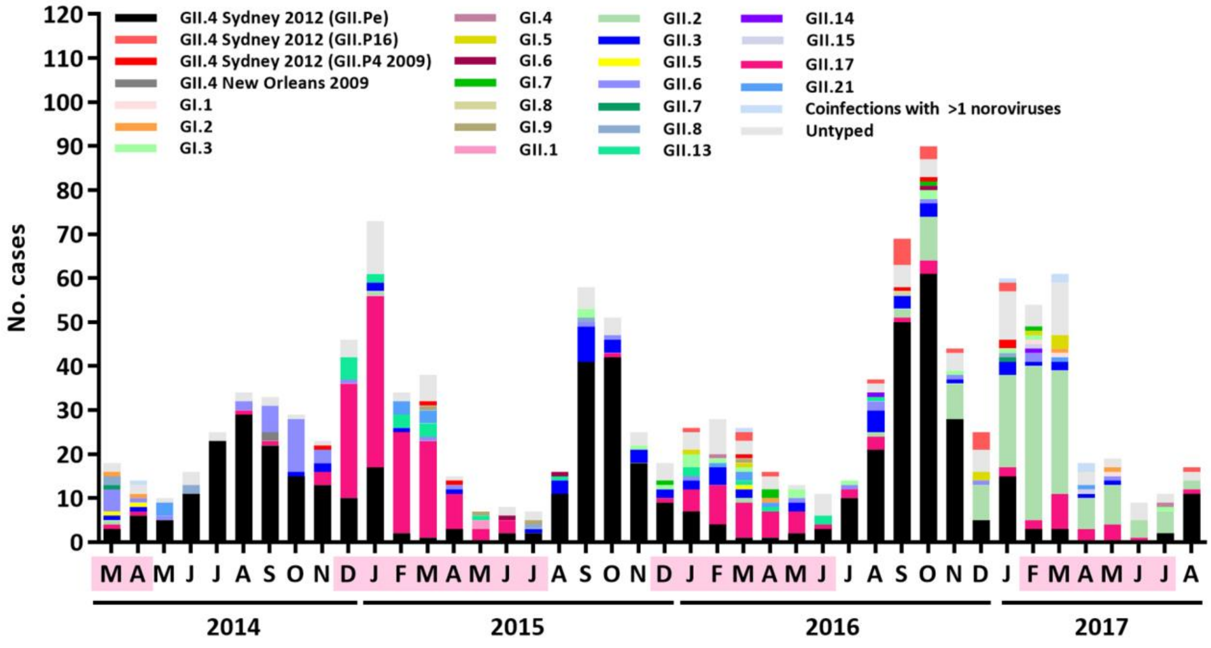

Technical Appendix Figure 1. Epidemic curve showing bimodal seasonality and alternating predominance of norovirus GII.4 and non-GII.4 (stratified by viral protein 1 genotype) in Hong Kong, China, 2014–2017. Pink shading along baseline indicates months during which the median age of hospitalized case-patients was >5 years as in Figure.

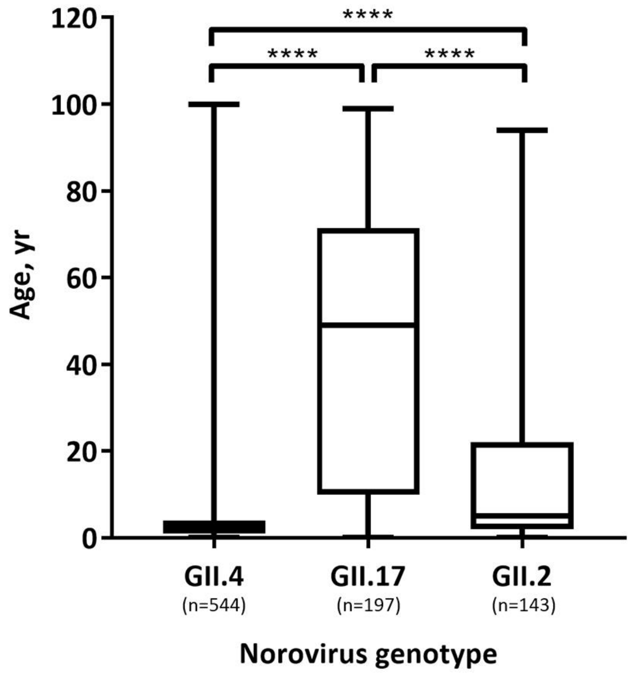

Technical Appendix Figure 2. Box-plot of age distribution of case-patients infected with norovirus viral protein 1 genotypes GII.4, GII.17, and GII.2. ****, two-tailed $p<0.0001$, as calculated by nonparametric Kruskal-Wallis test and corrected for Dunn's multiple comparisons test.
